# Endocrinologists Can Contribute to the Care of People Impacted by the Opioid Epidemic

**DOI:** 10.1210/clinem/dgz237

**Published:** 2019-11-28

**Authors:** Ann Danoff

**Affiliations:** Department of Medicine, Perelman School of Medicine, University of Pennsylvania, Philadelphia, PA

**Keywords:** opiates, opioids, hypogonadism, adrenal insufficiency, public health, endocrinopathy

Although the impact of opiates and opioids (naturally occurring and semi-synthetic/synthetic substances, respectively) on endocrine function has been recognized for decades (1), the meta-analysis by de Vries et al in this issue of *The Journal of Clinical Endocrinology & Metabolism* (2) sheds welcome light on the prevalence of hypogonadism (~63% in men) and adrenal insufficiency (~20% in both genders) among chronic opioid users. In view of the magnitude of the opioid epidemic and the prevalence of endocrine dysfunction in opioid users, this information is timely and offers an opportunity for endocrinologists to contribute to the care of a large number of people.

The number of individuals impacted by the opioid epidemic is sobering. According to the Centers for Disease Control (3), approximately 191 million prescriptions were written for opioids in 2017. Importantly, depending on definitions used (4), it is estimated that between 1% and 25% of people who receive a single prescription for opioids will develop opioid use disorder (OUD), suggesting that opioids prescribed with the intention of ameliorating acute pain can serve as a gateway drug to unhealthy prescription and/or illicit opioid use. The 2015 National Survey on Drug Use and Health (5) reported that in the United States (home to 4.27% of the world’s population and source of ~80% of the opioid prescriptions), approximately 92 million noninstitutionalized persons over the age of 12 years used prescription opioids, and it is estimated that approximately 4% of persons addicted to opioids will transition to heroin. In 2017, approximately 48000 deaths (130 deaths per day) have been attributed to prescription or nonprescription opioids. The death toll does not reflect the full spectrum of the devastation resulting from this crisis. It is estimated that 45% of opioid users experience a nonfatal overdose; jobs are lost, families destroyed, and communities devastated. Although there are racial differences (highest rates of OUD among whites) and geographic variation (3), no race, gender, socioeconomic class, or locale is spared.

How did we find ourselves in this predicament? In part, well-intentioned individuals recognized that as a health care community, we needed to be more sensitive to addressing our patients’ pain. But sadly, far more nefarious motivations fueled the opioid crisis. Van Zee (6) offers insight into how one major company engaged in systematic, aggressive promotional activities that included providing trinkets for physicians, profiling, and marketing to high-prescribing providers, offering lucrative incentives for sales representatives and free starter coupons for patients. Speakers’ bureaus were organized, and “thought leaders” who were designated and well-compensated to deliver talks marketed as “education” knowingly disseminated misinformation; an audience of colleagues were offered free trips to destination venues to “learn” from the anointed “experts.” In 2007, the highlighted company pled guilty to misbranding claims that their opioid product was less addictive and prone to less abuse and diversion. Many pharmaceutical companies continued to use similar marketing strategies, and despite the 2007 settlement, the rate of opioid prescriptions continued to rise and did not peak until 2012. This was all conducted in a setting in which there was no compelling evidence that opioids were an effective treatment for chronic pain (4, 7), and the suggestion that chronic opioid use might even result in hyperalgesia, thereby exacerbating the perception of pain.

The health care community played a significant role in propagating the epidemic (8). In an effort to address and mitigate pain, in 2001 the Joint Commission introduced pain as the “fifth vital sign,” the Institute of Medicine increased focus on patient satisfaction as a proxy for quality, and the Center for Medicare & Medicaid Services and the Agency for Healthcare Research and Quality created the Hospital Consumer of Healthcare Providers and Systems survey in which 3 of the 25 questions were related to pain control. In 2005, hospitals were rewarded with full compensation if they submitted the Hospital Consumer of Healthcare Providers and Systems survey data and incurred a 2% penalty if they did not. In 2010, as part of the Hospital Value Based-Purchasing Program, patient satisfaction (which emphasized attention to pain) was included as a component of payment incentives. In addition to deployment of system-wide metrics that encouraged opioid use, individual physicians were complicit. Some of us participated in marketing events, wrote prescriptions for opioids without sufficient consideration of the consequences, or did not recognize what was happening in our own backyard. An occasional physician participated in flagrant illegal activities related to opioids.

Much work is being directed toward addressing and mitigating the opioid epidemic with some success, although we still have a long way to go. The lay press is replete with public exposes of numerous pharmaceutical companies and chain drug stores implicated and the legal actions that have been taken against them. Some compensation has been collected, but it is important to note that settlements thus far represent a tiny fraction of the profits accrued from the sale of opioids and only approximately 3% of the 17 billion dollars predicted necessary to address the damages caused by the opioid epidemic. In addition to civil charges, criminal charges and charges against individuals believed responsible are underway. The Center for Medicare & Medicaid Services revised its recommendations for pain management in 2016 (7), and medically assisted treatment programs are available (although more are needed). Prescribing practices appear to be changing, with the annual prescribing rate decreased by 19% (3) between 2006 and 2017, and state surveillance programs have been introduced. Nonpharmacologic therapies (meditation, exercise, massage, etc), and non-opioid analgesics are now recommended for acute pain when possible (4,7). Attention is being directed to the number of pills dispensed and dose prescribed, with strong recommendations to prescribe lower doses where reasonable (higher doses are associated with higher rates of OUD).

What is the role of the endocrinologist in this context? The magnitude of several endocrine complications suggested in the meta-analysis in this issue of *The Journal of Clinical Endocrinology & Metabolism* offer the important opportunity for endocrinologists to be proactive in educating colleagues who utilize opioids to consider and evaluate their patients for these less well-appreciated endocrine complications associated with opioid use, to replace deficient hormones as appropriate, and consult with us as needed. It is also an obvious invitation for clinician investigators to explore other opiate/endocrine interactions. These might include expansion of work already being done by our community, including investigating the impact of opiates on the gonadal axis in women, other endocrine axes, the effect of replacement of deficiencies (on libido, bone health, fertility, recidivism, depression, hyperalgesia, quality of life, quality of end-of-life, etc), and rigorous comparison of endocrine effects of different opioids, to name just a few. We should collaborate on relevant guidelines and lend our expert opinion (until robust evidence is available). As physicians, we should also encourage and support further investigation into the biology, psychology, and social determinants of addiction and pain.

Is there a moral to this story? In addition to providing important information that should inform clinical care and research, we would be remiss if we did not engage in some self-reflection. It is fair to say we all share some responsibility for the opioid epidemic. We must learn from the history of this epidemic (and the analogous public health consequences associated with tobacco use) and work together to identify and overturn misinformation campaigns. This is the time to take off our rose-colored glasses and accept the hard lesson that not everyone is motivated by altruism. It is always helpful to review the core values of our profession to “do no harm” and the cautions in the Oath of Maimonides not to permit “avarice or miserliness” to drive our actions. We must make sure we partner only with those who share those values and remind ourselves that as physicians and scientists, we have an obligation to question, educate, and advance the health and well-being of our fellow humans.

## Abbreviation

OUD: opioid use disorder

## Additional Information

***Disclosure Summary:*** The author has nothing to disclose.
